# Risk factor analysis and nomogram establishment and verification of brain astrocytoma patients based on SEER database

**DOI:** 10.1038/s41598-023-33537-w

**Published:** 2023-05-12

**Authors:** Ruiqi Wang, Jiaxue Cui, Yizhuo Diao, Chenxin Jin, Yongxing Chen, Xiupeng Lv, Xiaofeng Li

**Affiliations:** 1 Department of Radiation Oncology, First Affiliated Hospital, Dalian Medical University, Dalian Liaoning, 116044 China; 2 Department of Epidemiology and Health Statistics, Dalian Medical University, 9 Lvshun South Road, Liaoning Dalian, 116044 China

**Keywords:** Diseases, Medical research, Oncology, Risk factors

## Abstract

Astrocytoma is a common brain tumor that can occur in any part of the central nervous system. This tumor is extremely harmful to patients, and there are no clear studies on the risk factors for astrocytoma of the brain. This study was conducted based on the SEER database to determine the risk factors affecting the survival of patients with astrocytoma of the brain. Patients diagnosed with brain astrocytoma in the SEER database from 2004 to 2015 were screened by inclusion exclusion criteria. Final screened brain astrocytoma patients were classified into low grade and high grade according to WHO classification. The risk factors affecting the survival of patients with low-grade and high-grade brain astrocytoma were analyzed by univariate Kaplan–Meier curves and log-rank tests, individually. Secondly, the data were randomly divided into training set and validation set according to the ratio of 7:3, and the training set data were analyzed by univariate and multivariate Cox regression, and the risk factors affecting the survival of patients were screened and nomogram was established to predict the survival rates of patients at 3 years and 5 years. The area under the ROC curve (AUC value), C-index, and Calibration curve are used to evaluate the sensitivity and calibration of the model. Univariate Kaplan–Meier survival curve and log-rank test showed that the risk factors affecting the prognosis of patients with low-grade astrocytoma included Age, Primary site, Tumor histological type, Grade, Tumor size, Extension, Surgery, Radiation, Chemotherapy and Tumor number; risk factors affecting the prognosis of patients with high-grade astrocytoma include Age, Primary site, Tumor histological type, Tumor size, Extension, Laterality, Surgery, Radiation, Chemotherapy and Tumor number. Through Cox regression, independent risk factors of patients with two grades were screened separately, and nomograms of risk factors for low-grade and high-grade astrocytoma were successfully established to predict the survival rate of patients at 3 and 5 years. The AUC values of low-grade astrocytoma training set patients were 0.829 and 0.801, and the C-index was 0.818 (95% CI 0.779, 0.857). The AUC values of patients in the validation set were 0.902, 0.829, and the C-index was 0.774 (95% CI 0.758, 0.790), respectively. The AUC values of high-grade astrocytoma training set patients were 0.814 and 0.806, the C-index was 0.774 (95% CI 0.758, 0.790), the AUC values of patients in the validation set were 0.802 and 0.823, and the C-index was 0.766 (95% CI 0.752, 0.780), respectively, and the calibration curves of the two levels of training set and validation set were well fitted. This study used data from the SEER database to identify risk factors affecting the survival prognosis of patients with brain astrocytoma, which can provide some guidance for clinicians.

## Introduction

Brain tumors refer to abnormal proliferation of cells in the brain and are the most common malignant tumors of the central nervous system^1,2^. Clinically divided into primary brain tumors and metastatic brain tumors^3^. Primary brain tumors refer to tumors caused by cells in the central nervous system, primary brain tumors account for about 1% of new cancers in the United States, about 2% of dead cancers in the United States, and their main primary tumor is glioma^4^. Previous studies have also found that brain tumors in childhood have a great impact on both morbidity and mortality in children^5^. At present, the traditional method of clinical treatment of brain tumors is surgery, radiotherapy and chemotherapy^6–8^. Astrocytoma, an aggressive tumor with the worst prognosis, can slightly improve survival with reasonable treatment, but the risk factors for this tumor have rarely been clearly studied^9^. This study used data from the U.S. National Public Database to analyze risk factors affecting the survival of patients with brain astrocytoma. The SEER database is currently the largest public cancer database, covering approximately 28% of the U.S. population, and the SEER database includes basic information about the U.S. population and information about relevant cancer characteristics^10^.

In recent years, nomogram have been widely used in the prediction of various diseases, especially tumors. It meets the needs of integrated models and plays a very important role in the current "digital medicine" environment, using nomogram to facilitate prognosis predictions for clinicians^11–13^. Therefore, this study aims to use the data from the SEER database to screen for risk factors affecting the survival of patients with brain astrocytoma, and to establish nomogram model of the survival rate of patients at 3 and 5 years, so as to guide doctors in predicting the prognosis of patients and provide assistance to clinicians.

## Materials and methods

### Data source

The data for this study were selected from the SEER database established by the National Cancer Institute, and we selected the database containing 13 registries with radiotherapy data, which provided data that could support the completion of this study. A total of 6154 patients diagnosed with astrocytoma of the brain from 2004 to 2015 were extracted from the database, and a total of 2214 patients were screened according to the inclusion and exclusion criteria. The types of astrocytoma include diffuse astrocytoma, anaplastic astrocytoma, pilocytic astrocytoma, unique astrocytoma variants, and astrocytoma, NOS above five types.

### Inclusion and exclusion criteria

#### Inclusion criteria

(i) Patients with astrocytoma of the brain diagnosed in 2004–2015; (ii) The international tumor code ICD-0-3 is C70.0-C75.3, including the brain, frontal lobe, parietal lobe, temporal lobe, occipital lobe, etc. (iii) Those with complete clinical information.

#### Exclusion criteria

(i) Baseline information (e.g., race) is unknown; (ii) tumor size and tumor number are missing; (iii) survival time is unknown; (iv) proven only at autopsy or death.

### Grouping methods

For a more intuitive and standardized study, the study data were transformed into dichotomous or multi-categorical variables. Age was classified into five age groups: < 20, 20–39, 40–59, 60–79, and ≥ 80; Race into black, white, and others; and Histological type into five categories: diffuse astrocytoma, anaplastic astrocytoma, pilocytic astrocytoma, unique astrocytoma variants, and astrocytoma, NOS. The Primary site was divided into brain (C71.0–C71.5), cerebellum (C71.6), brainstem (C71.7), spinal cord (C72.0), and others (C70.0 71.8 C71.9 C72.3 C72.5 C72.8 C75.1 C75.3); the Lateral division was unilateral and bilateral; the Grade was I-IV; for continuous variable Tumor size and Extension were divided using X-tile to select the best grouping method, and finally the Tumor size was classified as ≤ 60 mm and ≥ 61 mm, and the Extension was classified as 10–30 mm and 40–75 mm; Surgery, Radiation, Chemotherapy: yes/no; Tumor number was grouped as 1 and > 1.

### Statistical methods

The data extracted from the SEER database were first organized according to the inclusion and exclusion criteria using Excel and classified into low-grade and high-grade brain astrocytoma patients according to WHO classification. The survival rates were calculated by Kaplan–Meier curve method using R-studio 4.2.2 software for low-grade and high-grade brain astrocytoma patients, respectively. and the effect of the included factors on patient survival was demonstrated by K–M curves, and log-rank test was used for group comparisons of the same variables. The data of low-grade and high-grade astrocytoma were randomly divided into training set and validation set in a 7:3 ratio with R-studio software, and χ^2^ tests were performed between different variables in the training and validation sets using SPSS. Univariate and multivariate Cox regression analyses were performed on the training set data using R-studio4.1.1, create a nomogram of the final filtered variables using the R packages 'rms', 'foreign', and 'survival', and the area under the ROC curve (AUC value) and C-index were used to evaluate the accuracy of the model, with AUC and C-index taking values ranging from 0–1, the closer to 1 indicating the more accurate the model; the calibration curve was used to evaluate the calibration degree of the model, and the closer the calibration curve was to the standard curve indicating the stronger predictive ability of the model. The differences were considered statistically significant at P < 0.05, except for the univariate Cox regression at P < 0.1.

## Results

### Comparison of patient baseline features

In this study, a total of 2214 patients were included in the study, there were 539 patients with low-grade astrocytoma and 1675 patients with high-grade astrocytoma. R-studio 4.2.2 was randomly split into training set and validation set according to the ratio of 7:3, with 379 patients in the low-level training set, 160 patients in the validation set, 1175 patients in the high-level training set, and 500 patients in the validation set. Comparing the different variables in the training set and the validation set, the p-value of the χ^2^ test result was > 0.05, and the difference was not statistically significant, indicating that the two groups were randomly assigned. Information on the two different grades and the results of the χ^2^ tests are shown in Tables 1 and 2.

**Table 1:** General data on training and validation sets for patients with low-grade astrocytoma n (%).

Variables	Training set (n = 379)	Validation set (n = 160)	P
Age			
< 20	133 (35.1)	45 (28.1)	0.343
20–39	114 (30.1)	50 (31.2)	
40–59	90 (23.7)	42 (26.3)	
60–79	36 (9.5)	18 (11.3)	
≥ 80	6 (1.6)	5 (3.1)	
Sex			
Male	200 (52.8)	93 (58.1)	0.258
Female	179 (47.2)	67 (41.9)	
Race			
Black	31 (8.2)	18 (11.2)	0.254
White	317 (83.6)	134 (83.8)	
Others	31 (8.2)	8 (5.0)	
Histology type			
Diffuse	27 (7.1)	14 (8.7)	0.283
Anaplastic	12 (3.2)	7 (4.4)	
Pilocytic	149 (39.3)	48 (30.0)	
Unique	12 (3.2)	8 (5.0)	
NOS	179 (47.2)	83 (51.9)	
Primary site			
Brain	212 (55.9)	106 (66.2)	0.079
Cerebellum	67 (17.7)	14 (8.8)	
Brainstem	30 (7.9)	11 (6.9)	
Spinal cord	14 (3.7)	7 (4.4)	
Others	56 (14.8)	22 (13.7)	
Laterality			
Bilateral	9 (2.4)	4 (2.5)	1.000
Unilateral	370 (97.6)	156 (97.5)	
Grade			
I	143 (37.7)	52 (32.5)	0.281
II	236 (62.3)	108 (67.5)	
Tumor size (mm)			
≤ 60	323 (85.2)	144 (90.0)	0.166
≥ 61	56 (14.8)	16 (10.0)	
Extension (mm)			
10–30	349 (92.1)	141 (88.1)	0.400
40–75	30 (7.9)	19 (11.9)	
Surgery			
Yes	302 (79.7)	127 (79.4)	1.000
No	77 (20.3)	33 (20.6)	
Radiotherapy			
Yes	104 (27.4)	53 (33.1)	0.213
No	275 (72.6)	107 (66.9)	
Chemotherapy			
Yes	62 (16.4)	35 (21.9)	0.141
No	317 (83.6)	125 (78.1)	
Tumor number			
1	353 (93.1)	143 (89.4)	0.164
> 1	26 (6.9)	17 (10.6)

**Table 2:** General data on training and validation sets for patients with high-grade astrocytoma n (%).

Variables	Training set (n = 1175)	Validation set (n = 500)	P
Age			
< 20	78 (6.6)	23 (4.6)	0.329
20–39	309 (26.3)	146 (29.2)	
40–59	383 (32.6)	172 (34.4)	
60–79	346 (29.5)	135 (27.0)	
≥ 80	59 (5.0)	24 (4.8)	
Sex			
Male	642 (54.6)	275 (55.0)	0.915
Female	533 (45.4)	225 (45.0)	
Race			
Black	85 (7.2)	24 (4.8)	0.133
White	975 (83.0)	432 (86.4)	
Others	115 (9.8)	44 (8.8)	
Histology type			
Diffuse	78 (6.6)	25 (5.0)	0.043
Anaplastic	909 (77.4)	420 (84.0)	
Pilocytic	12 (1.0)	4 (0.8)	
Unique	19 (1.6)	7 (1.4)	
NOS	157 (13.4)	44 (8.8)	
Primary site			
Brain	902 (76.8)	392 (78.4)	0.556
Cerebellum	38 (3.2)	12 (2.4)	
Brainstem	34 (2.9)	13 (2.6)	
Spinal cord	13 (1.1)	2 (0.4)	
Others	188 (16.0)	81 (16.2)	
Laterality			
Bilateral	15 (1.3)	11 (2.2)	0.194
Unilateral	1160 (98.7)	489 (97.8)	
Grade			
III	102 (8.7)	27 (5.4)	0.021
IV	1073 (91.3)	473 (94.6)	
Tumor size (mm)			
≤ 60	951 (80.9)	413 (82.6)	0.450
≥ 61	224 (19.1)	87 (17.4)	
Extension (mm)			
10–30	960 (81.7)	403 (80.6)	0.631
40–75	215 (18.3)	97 (19.4)	
Surgery			
Yes	836 (71.1)	346 (69.2)	0.446
No	339 (28.9)	154 (30.8)	
Radiotherapy			
Yes	925 (78.7)	403 (80.6)	0.429
No	250 (21.3)	97 (19.4)	
Chemotherapy			
Yes	801 (68.2)	349 (69.8)	0.527
No	374 (31.8)	151 (30.2)	
Tumor number			
1	1039 (88.4)	433 (86.6)	0.326
> 1	136 (11.6)	67 (13.4)	

### Impact of different factors on patient survival

#### Risk factor analysis affecting survival in patients with low-grade astrocytoma

**Figure 1:**
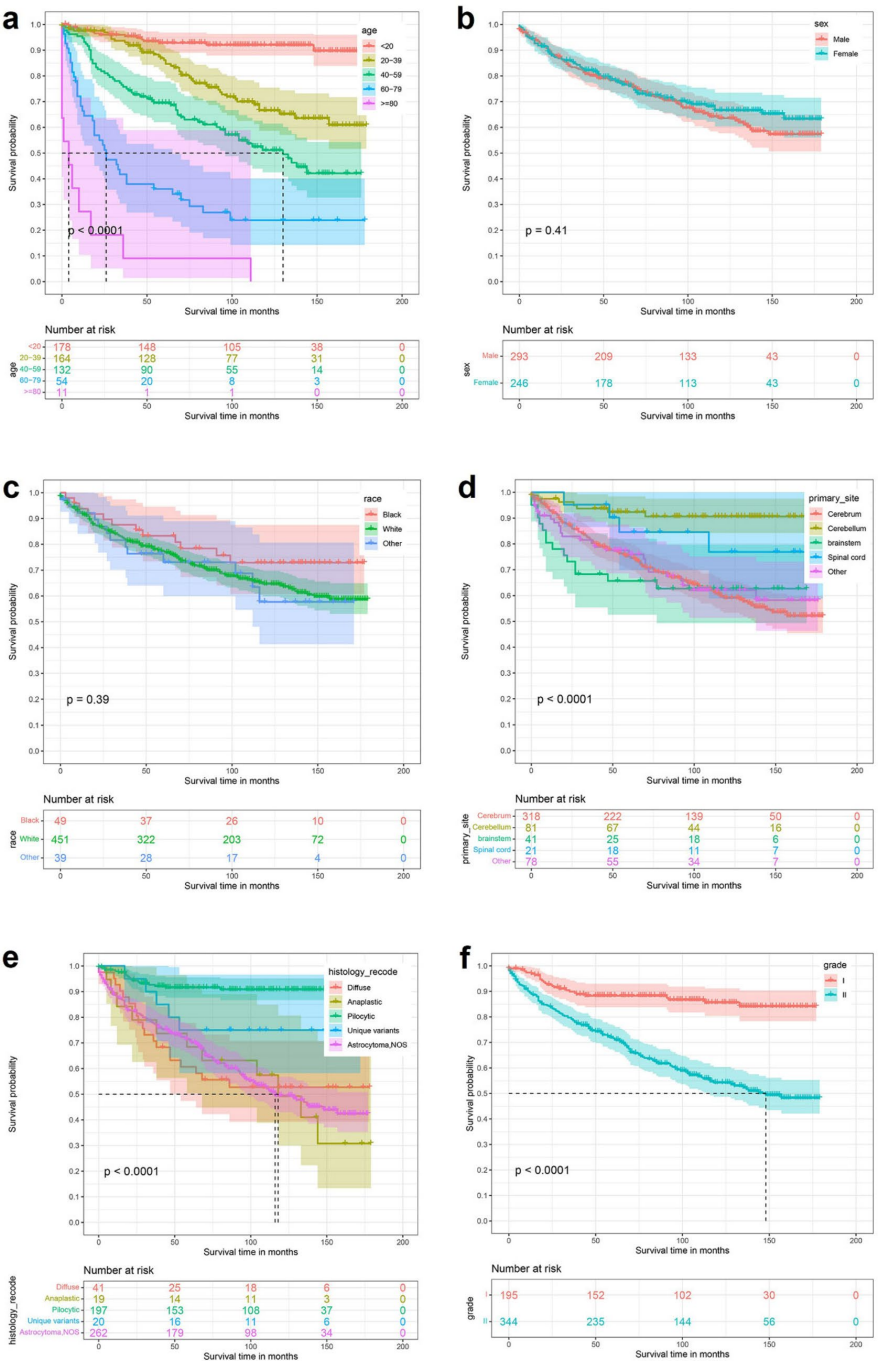

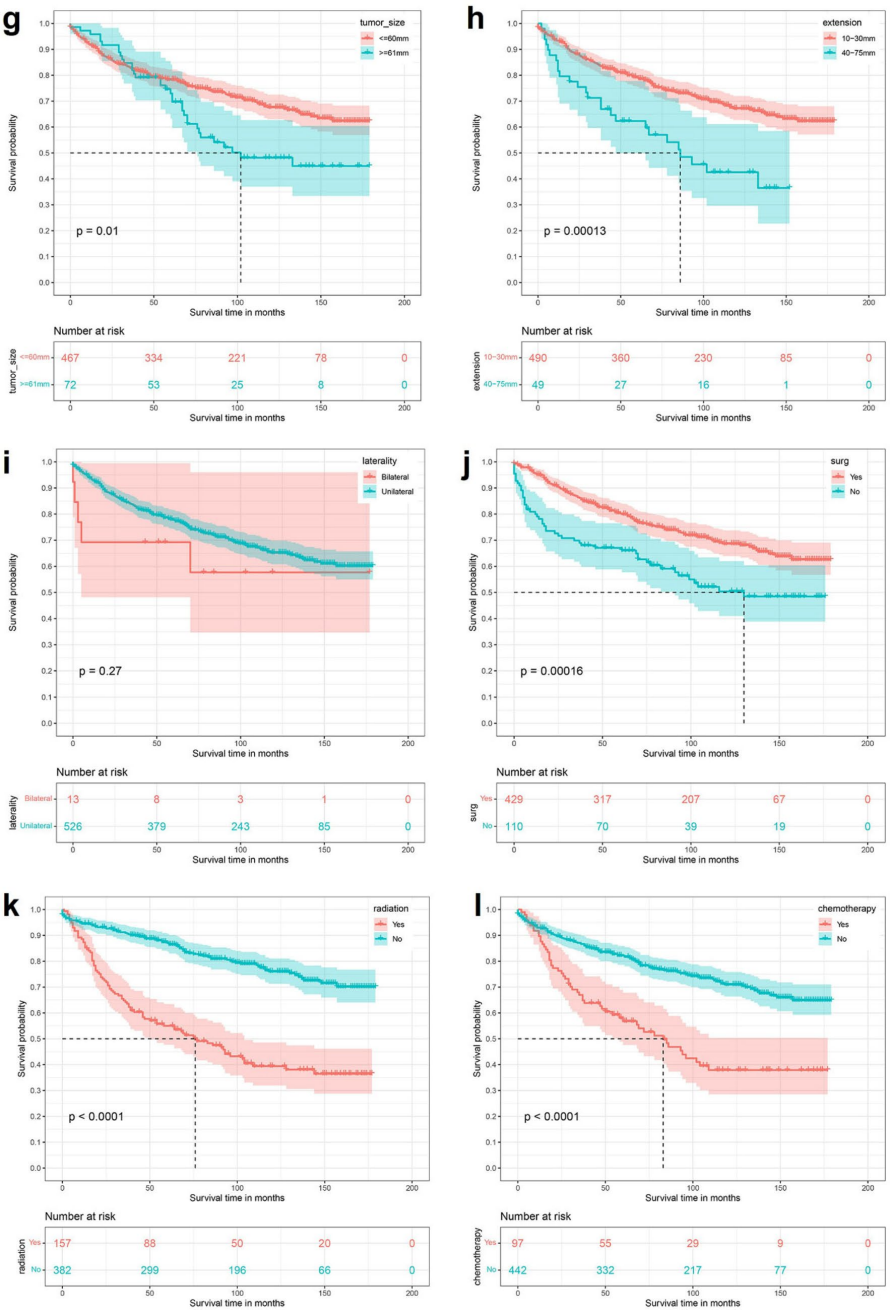

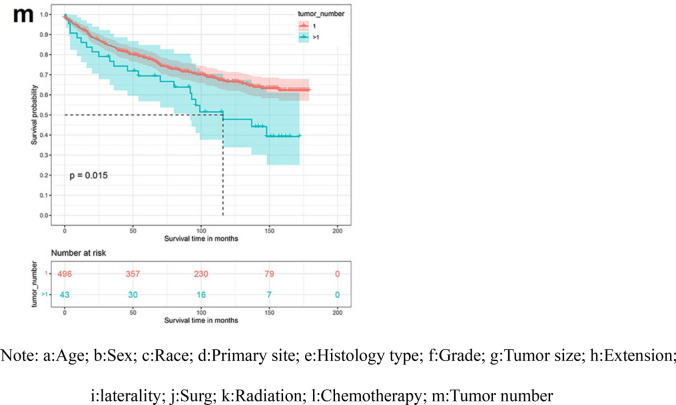
Kaplan Meier survival curve in low-grade astrocytoma patients. (**a**) Age; (**b**) sex; (**c**) race; (**d**) primary site; (**e**) histology type; (**f**) grade; (**g**) tumor size; (**h**) extension; (**i**) laterality; (**j**) Surg; (**k**) radiation; (**l**) chemotherapy; (**m**) tumor number.

By univariate Kaplan–Meier survival curve and log-rank test, Age (P < 0.0001), Primary site (P < 0.0001), Tumor histological type (P < 0.0001), Tumor size (P = 0.01), Extension (P = 0.00013), Surgery (P = 0.00016), Radiation (P < 0.0001), Chemotherapy (P < 0.0001) and Tumor number (P = 0.015) were risk factors affecting the prognosis of patients with low-grade astrocytoma. The established K-M survival curve and log-rank test results showed that the factors of Age ≥ 80 years, Primary site at brainstem, Diffuse astrocytoma, Tumor ≥ 61 mm, deeper Extension, Bilateral, no Surgery, Radiotherapy, Chemotherapy and Tumor number > 1 were all related to poor survival time (Fig. 1).

#### Risk factor analysis affecting survival in patients with high-grade astrocytoma

**Figure 2:**
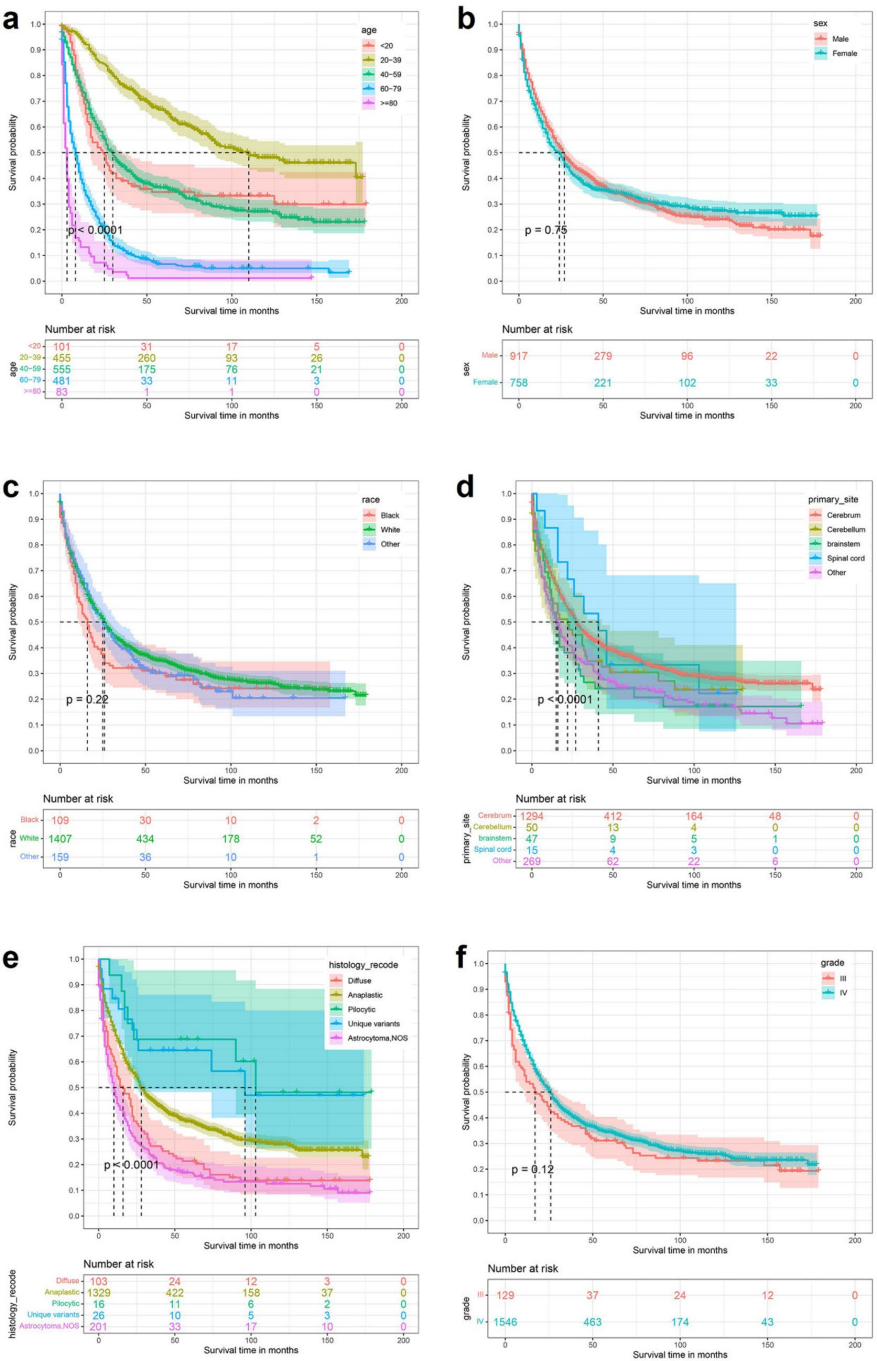

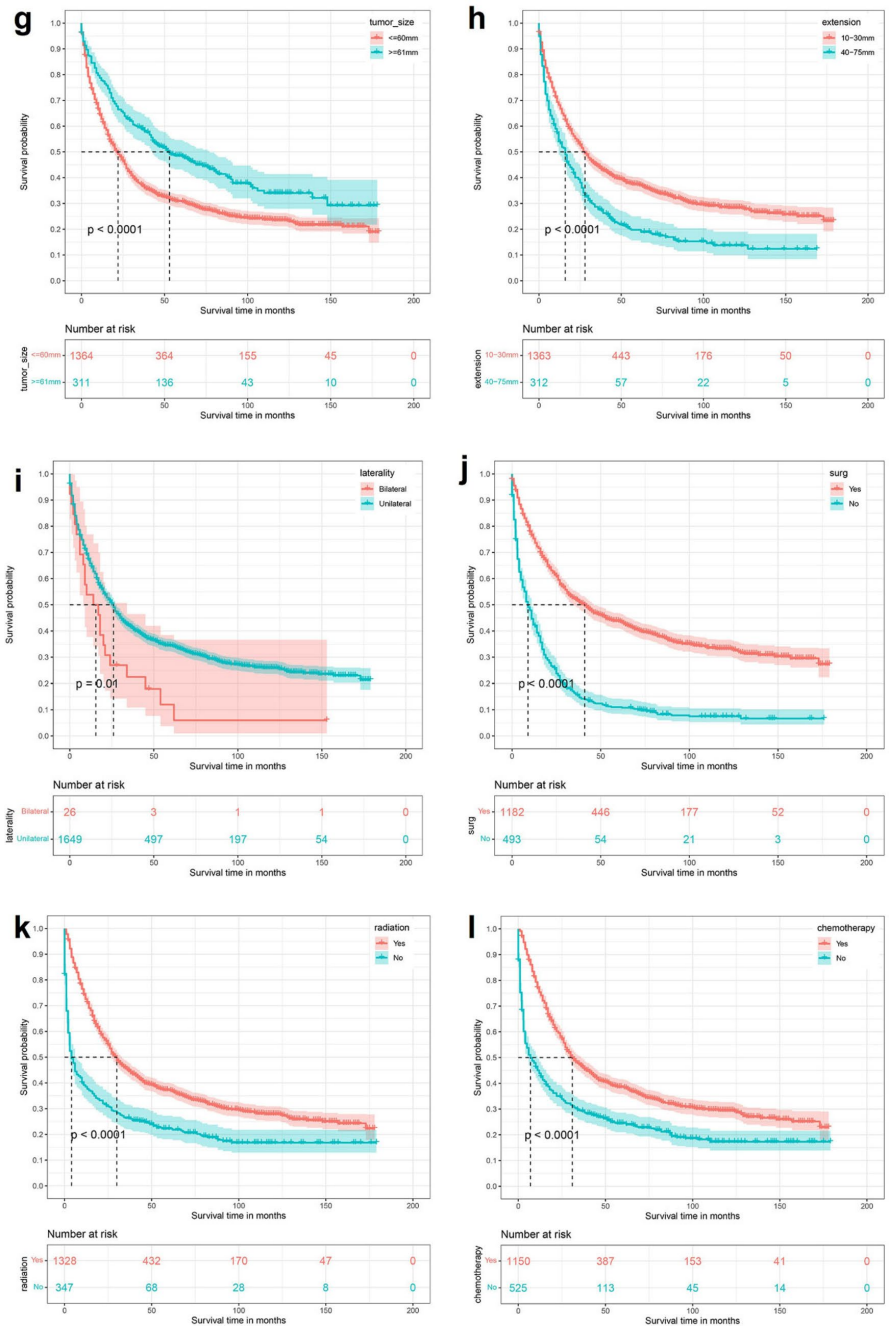

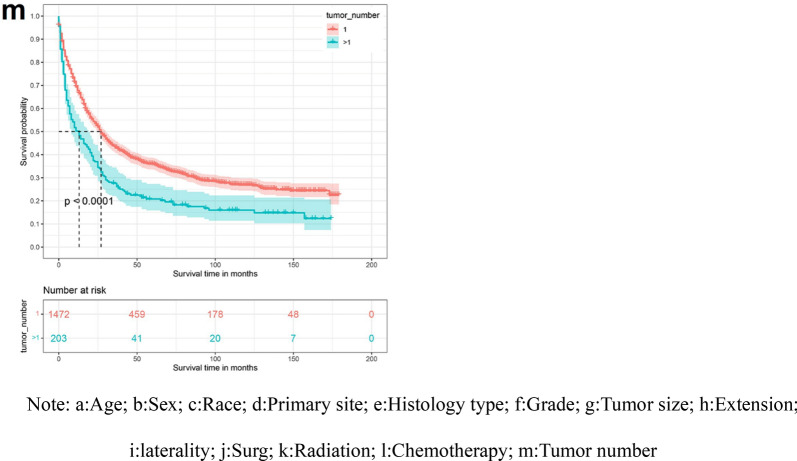
Kaplan Meier survival curve in high-grade astrocytoma patients. (**a**) Age; (**b**) sex; (**c**) race; (**d**) primary site; (**e**) histology type; (**f**) grade; (**g**) tumor size; (**h**) extension; (**i**) laterality; (**j**) Surg; (**k**) radiation; (**l**) chemotherapy; (**m**) tumor number.

By univariate Kaplan–Meier survival curve and log-rank test, Age (P < 0,0001), Primary site (P < 0.0001), Tumor histology type (P < 0.0001), Tumor size (P < 0.0001), Extension (P < 0.0001), Laterality (P = 0.01), Surgery (P < 0.0001), Radiation (P < 0.0001), Chemotherapy (P < 0.0001) and Tumor number (P < 0.0001) are risk factors for the prognosis of patients with high-grade astrocytoma. The results of the established K-M survival curves and log-rank tests showed that Age ≥ 80 years, Primary site at brainstem, Astrocytoma, NOS, Tumor size < 60 mm, deeper Extension, Bilateral, no Surgery, no Radiotherapy or Chemotherapy and Tumor number > 1 were all associated with poorer survival time in patients (Fig. 2).

### Single-factor and multi-factor Cox regression results

#### Univariate and multivariate COX regression results for low-grade astrocytoma

Patient data from the low-grade astrocytoma training set (13 variables) were included in univariate Cox regression analysis, and the univariate Cox regression excluded the gender variable (P > 0.1). To avoid omitting important variables, 12 variables with P < 0.1 in the univariate Cox regression were included in the multivariate Cox regression. If P < 0.1 in the univariate Cox regression analysis, the factor was associated with prognostic survival of the patients; if P < 0.05 in the multivariate Cox regression analysis, the factor was an independent factor affecting the survival of the patients. The univariate Cox regression results of this study showed that age greater than 40 years, white ethnicity, histological type of tumor, primary site, lateral bilateral tumor, grade II, larger tumor size, deeper entry into the brain, surgery, radiotherapy, chemotherapy, and the number of tumors were factors related to the prognosis and survival of patients; multivariate Cox regression results showed that older age, bilateral tumors, and radiotherapy and chemotherapy were independent factors affecting patient survival (Table 3).

**Table 3:** Univariate and multivariate Cox regression analysis of low-grade astrocytoma training set patient survival.

Variables	Univariate analysis (n = 539)		Multifactorial analysis (n = 539)	
	HR (95% CI)	P	HR (95% CI)	P
Age				
< 20	Ref	Ref	Ref	Ref
20–39	6.964 (3.109–15.600)	0.141	4.320 (1.785–10.457)	0.001
40–59	10.060 (4.505–22.460)	< 0.0001	5.604 (2.244–13.996)	0.000
60–79	22.258 (9.567–51.780)	< 0.0001	17.924 (6.989–45.965)	< 0.0001
≥ 80	58.977 (19.694–176.620)	< 0.0001	35.820 (10.638–120.611)	< 0.0001
Sex				
Male	Ref	Ref		
Female	0.903 (0.625–1.305)	0.588		
Race				
Black	Ref	Ref	Ref	Ref
White	3.016 (1.111–8.190)	0.030	1.912 (0.409–3.475)	0.748
Others	2.043 (0.598–6.980)	0.255	1.354 (0.362–5.067)	0.652
Histology type				
Diffuse	Ref	Ref	Ref	Ref
Anaplastic	1.574 (0.599–4.136)	0.358	0.838 (0.306–2.296)	0.730
Pilocytic	0.190 (0.080–0.446)	0.000	0.640 (0.237–1.731)	0.379
Unique	0.594 (0.163–2.159)	0.429	1.121 (0.294–4.269)	0.867
NOS	1.467 (0.761–2.820)	0.252	1.457 (0.740–2.865)	0.276
Primary site				
Brain	Ref	Ref	Ref	Ref
Cerebellum	0.133 (0.049–0.362)	< 0.0001	0.829 (0.268–2.559)	0.744
Brainstem	0.768 (0.386–1.529)	0.453	2.182 (0.954–4.988)	0.064
Spinal cord	0.312 (0.077–1.268)	0.103	0.370 (0.087–1.567)	0.177
Others	0.749 (0.444–1.262)	0.278	0.757 (0.411–1.392)	0.370
Laterality				
Bilateral	Ref	Ref	Ref	Ref
Unilateral	0.414 (0.152–1.126)	0.084	0.151 (0.047–0.489)	0.002
Grade				
I	Ref	Ref	Ref	Ref
II	3.878 (2.317–6.491)	< 0.0001	1.752 (0.963–3.185)	0.066
Tumor size (mm)				
≤ 60 mm	Ref	Ref	Ref	Ref
≥ 61 mm	1.886 (1.225–2.906)	0.004	1.539 (0.953–02.486)	0.078
Extension				
10–30 mm	Ref	Ref	Ref	Ref
40–75 mm	1.729 (0.970–3.082)	0.064	1.213 (0.614–2.398)	0.579
Surgery				
Yes	Ref	Ref	Ref	Ref
No	1.804 (1.204–2.704)	0.004	1.054 (0.650–1.712)	0.830
Radiotherapy				
Yes	Ref	Ref	Ref	Ref
No	0.291 (0.202–0.420)	< 0.0001	0.554 (0.367–0.837)	0.005
Chemotherapy				
Yes	Ref	Ref	Ref	Ref
No	0.448 (0.295–0.680)	0.000	0.535 (0.322–0.888)	0. 015
Tumor number				
1	Ref	Ref	Ref	Ref
> 1	1.772 (0.975–3.224)	0.061	0.782 (0.408–1.499)	0.459

#### Univariate and multivariate COX regression results for high-grade astrocytoma

The results of high-grade astrocytoma univariate Cox regression showed that age greater than 60 years, diffuse astrocytoma, initial location, bilateral tumors, tumor size, deeper entry into the brain, surgery, radiotherapy, chemotherapy, and tumor number were factors related to the patient's prognosis and survival. Multivariate Cox regression results showed that older age, diffuse astrocytoma, initial location, bilateral tumor, tumor size, deeper brain penetration, surgery, radiotherapy, and chemotherapy were independent factors affecting patient survival (Table 4).

**Table 4:** Univariate and multivariate Cox regression analysis of high-grade astrocytoma training set patient survival.

Variables	Univariate analysis (n = 1675)		Multifactorial analysis (n = 1675)	
	HR (95% CI)	P	HR (95% CI)	P
Age				
< 20	Ref	Ref	Ref	Ref
20–39	0.458(0.330–0.635)	< 0.0001	0.582 (0.413–0.819)	0.002
40–59	1.053 (0.778–1.425)	0.737	1.243 (0.900–1.716)	0.187
60–79	2.947 (2.184–3.977)	< 0.0001	3.169 (2.284–4.397)	< 0.0001
≥ 80	6.398 (4.364–9.379)	< 0.0001	4.926 (3.244–7.481)	< 0.0001
Sex				
Male	Ref	Ref		
Female	0.938 (0.817–1.077)	0.364		
Race				
Black	Ref	Ref		
White	0.847 (0.653–1.099)	0.212		
Others	0. 973 (0.701–1.351)	0.869		
Histology type				
Diffuse	Ref	Ref	Ref	Ref
Anaplastic	0.668 (0.519–0.860)	0.002	0.785 (0.607–1.016)	0.066
Pilocytic	0.281 (0.113–0.698)	0.006	0.368 (0.146–0.931)	0.035
Unique	0.276 (0.127–0.602)	0.001	0.333 (0.151–0.736)	0.007
NOS	1.295 (0.967–1.736)	0.083	1.030 (0.761–1.393)	0.848
Primary site				
Brain	Ref	Ref	Ref	Ref
Cerebellum	1.218 (0.828–1.791)	0.316	1.044 (0.703–1.550)	0.831
Brainstem	1.365 (0.907–2.053)	0.135	1.655 (1.071–2.557)	0.023
Spinal cord	1.027 (0.550–1.919)	0.932	1.315 (0.693–2.494)	0.403
Others	1.486 (1.242–1.776)	< 0.0001	1.171 (0.974–1.409)	0.094
Laterality				
Bilateral	Ref	Ref	Ref	Ref
Unilateral	0.595 (0.351–1.010)	0.054	0.396 (0.230–0.681)	0.001
Grade				
III	Ref	Ref		
IV	0.862 (0.682–1.090)	0.215		
Tumor size (mm)				
≤ 60 mm	Ref	Ref	Ref	Ref
≥ 61 mm	0.645 (0.536–0.777)	< 0.0001	0.725 (0.596–0.881)	0.001
Extension				
10–30 mm	Ref	Ref	Ref	Ref
40–75 mm	1.583 (1.341–1.868)	< 0.0001	1.418 (1.188–1.692)	0.000
Surgery				
Yes	Ref	Ref	Ref	Ref
No	2.797 (2.422–3.230)	< 0.0001	1.581 (1.351–1.850)	< 0.0001
Radiotherapy				
Yes	Ref	Ref	Ref	Ref
No	1.998 (1.703–2.345)	< 0.0001	1.411 (1.147–1.736)	< 0.0001
Chemotherapy				
Yes	Ref	Ref	Ref	Ref
No	1.873 (1.624–2.161)	< 0.0001	1.554 (1.292–1.868)	< 0.0001
Tumor number				
1	Ref	Ref	Ref	Ref
> 1	1.612 (1.321–1.969)	< 0.0001	1.122 (0.912–1.381)	0.276

### Creation of nomogram

**Figure 3:**
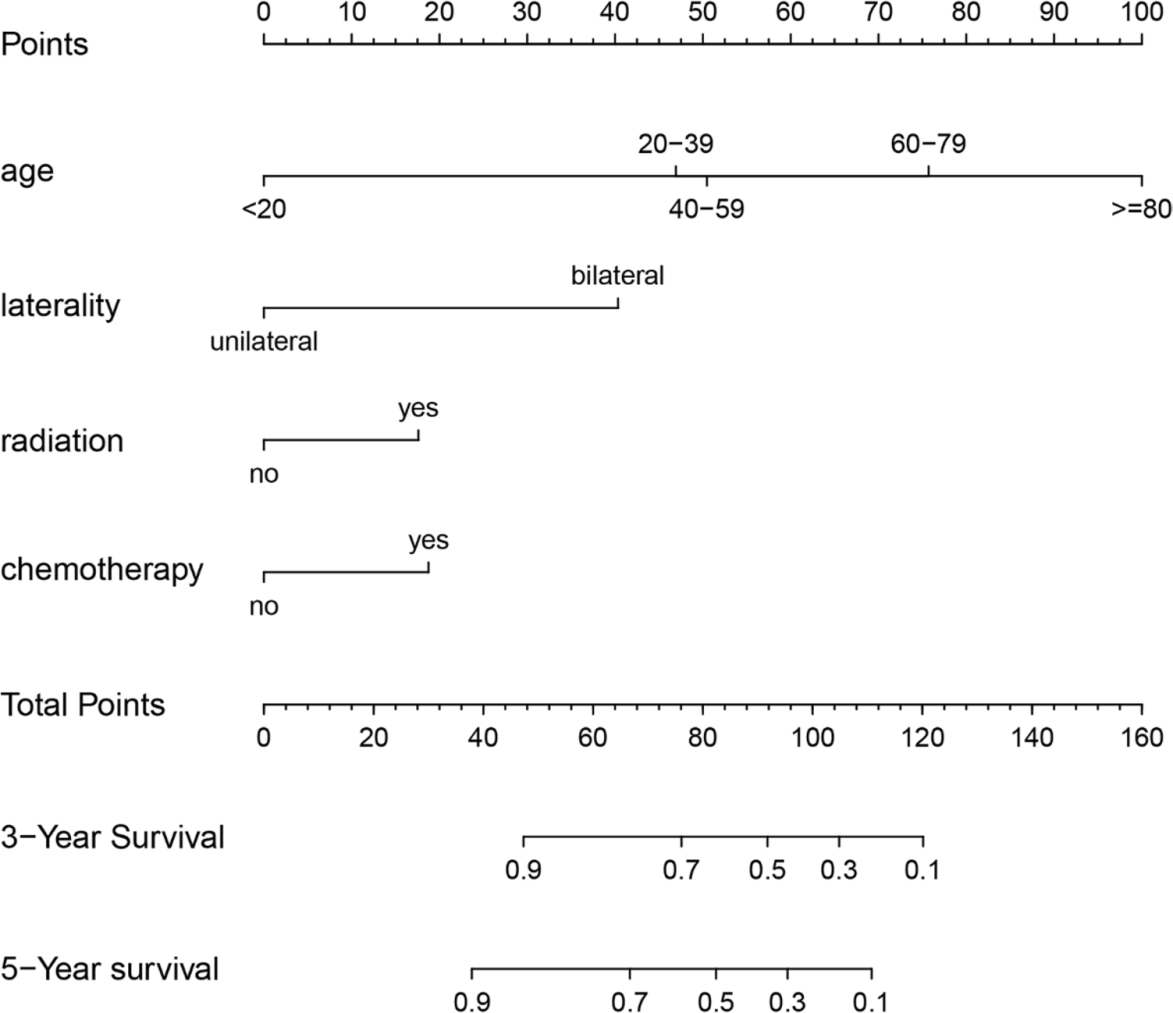
Nomogram of 3-year and 5-year survival prediction for patients with low-astrocytoma of the brain astrocytoma.

**Figure 4:**
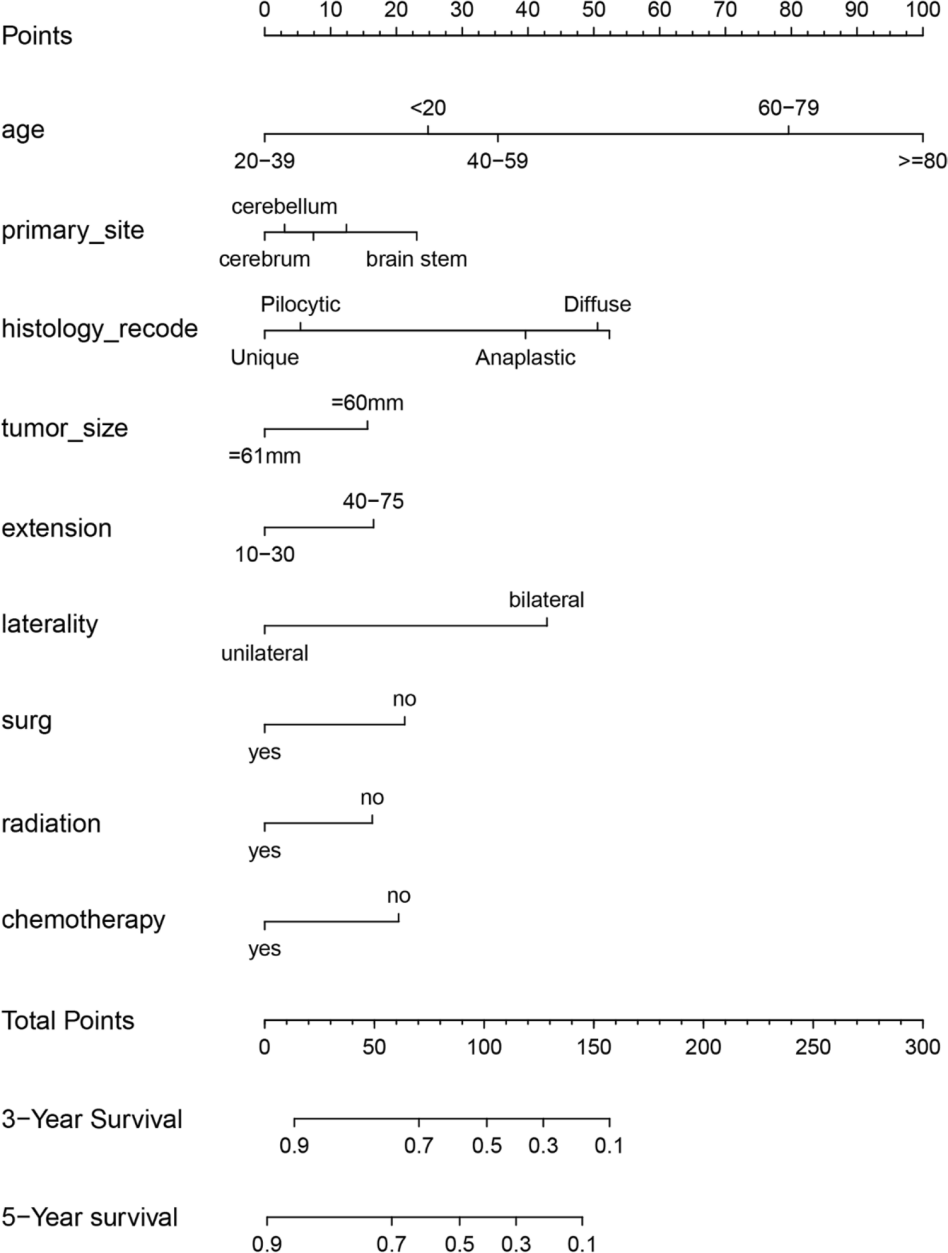
Nomogram of 3-year and 5-year survival prediction for patients with high-astrocytoma of the brain astrocytoma.

The variables screened in the multifactorial Cox regression analysis (P < 0.05) were included in the R-studio software to create a nomogram model. Different values for each variable were taken to obtain different values of scores, and the total scores were obtained by adding all the scores of each variable, and according to the total scores, the survival rate of patients at 3 and 5 years could be predicted accordingly (Figs. 3, 4).

### Validation of nomogram

The area under the ROC curve and C-index were used to evaluate the discrimination of the model, and the calibration curve was used to evaluate the calibration of the model.

#### Validation of nomogram in patients with low-grade astrocytoma

**Figure 5:**
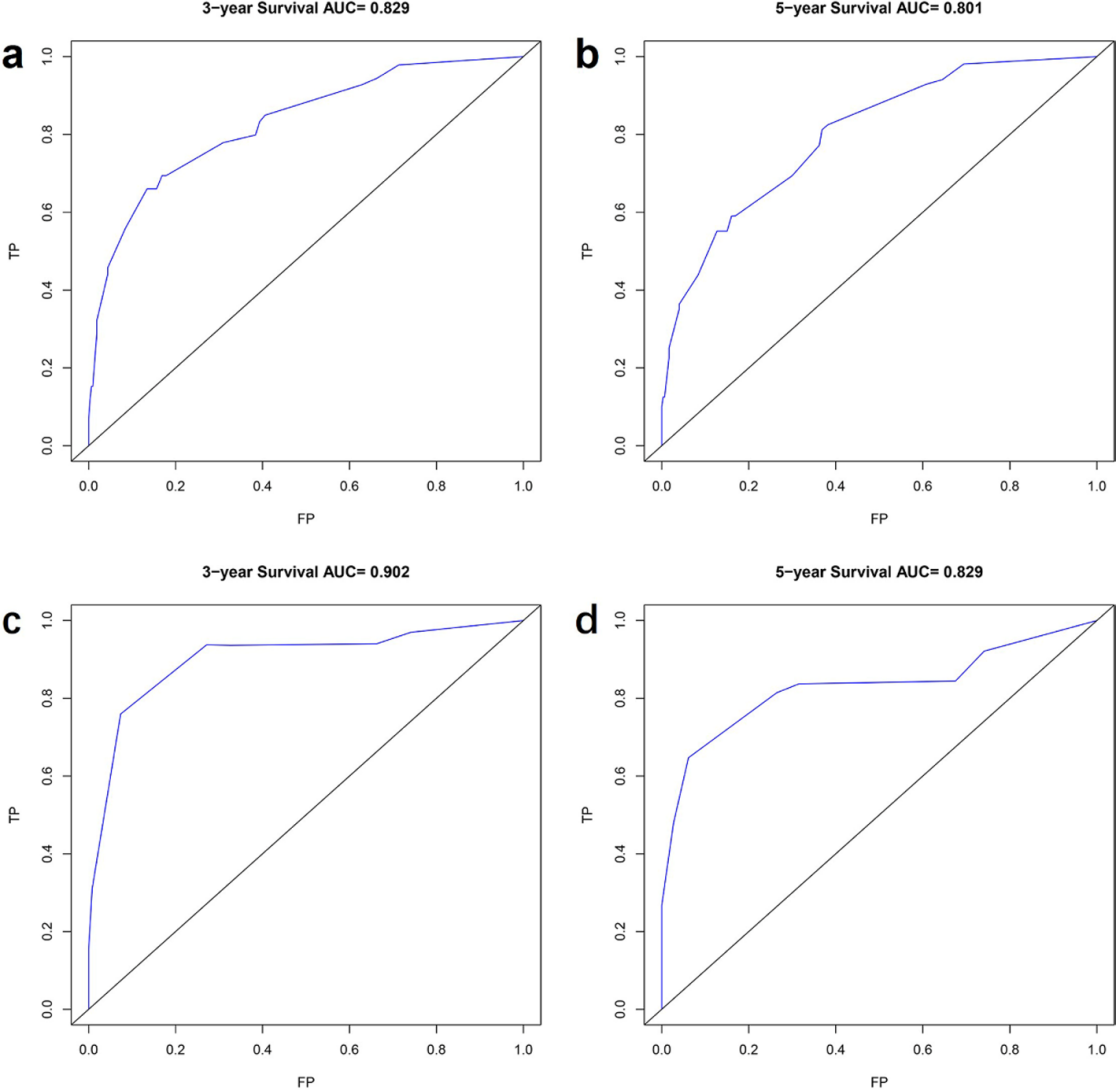
ROC curves of 3-year and 5-year survival prediction in patients with low-grade astrocytoma (**a** and **b** are the training set, **c** and **d** are the validation sets).

**Figure 6:**
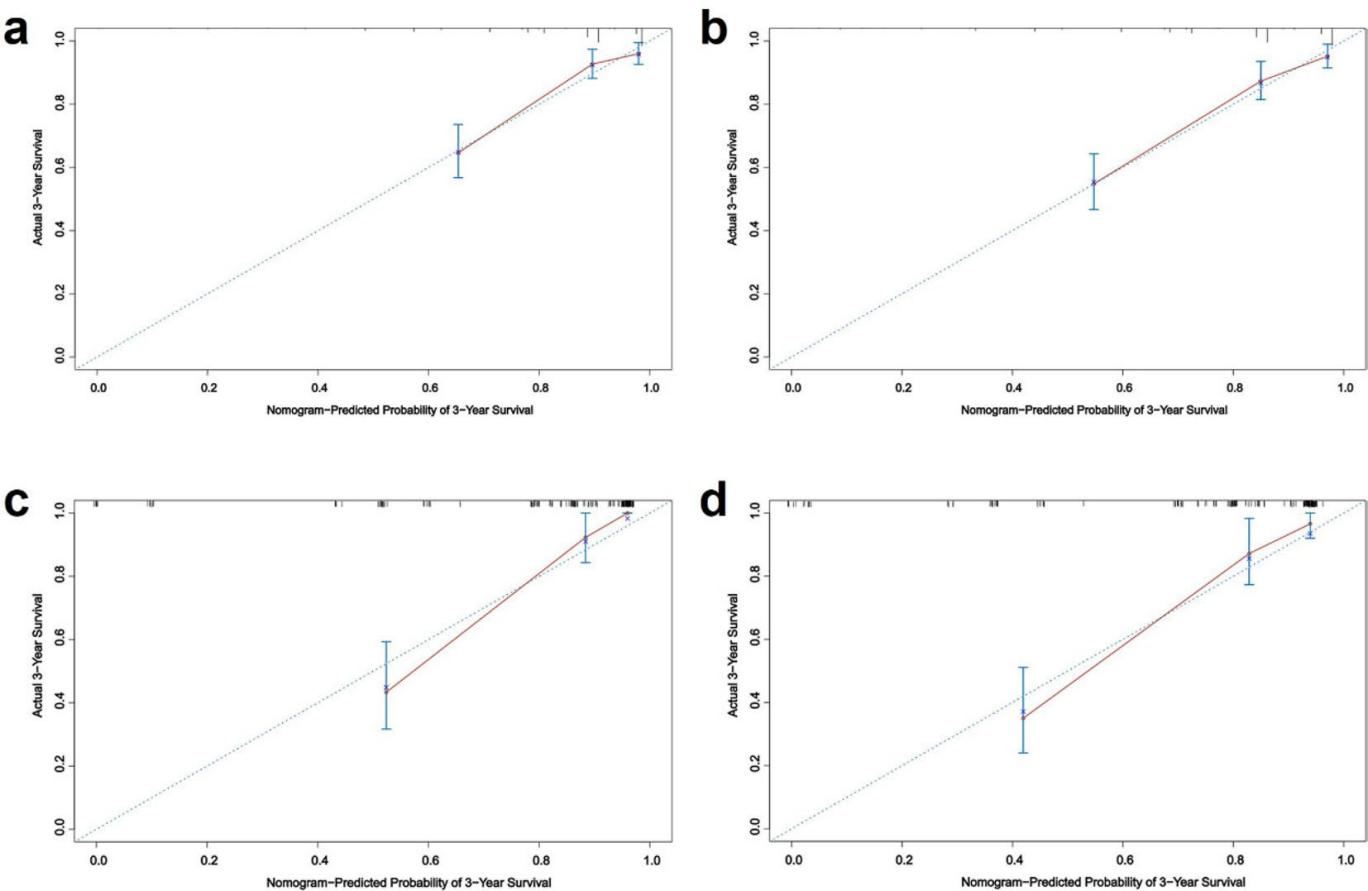
3-year and 5-year survival calibration curves for patients with low-grade astrocytoma (**a** and **b** are training sets, **c** and **d** are validation sets).

The AUC values of 3-year and 5-year survival rates of patients with low-grade astrocytoma training set were 0.829 and 0.801, respectively, and the AUC values of 3-year and 5-year survival rates of patients in the validation set were 0.902 and 0.829, respectively (Fig. 5). The C-index was 0.818 (95% CI 0.779, 0.857) for patients in the training set and 0.834 (95% CI 0.785, 0.883) for patients in the validation set. Meanwhile, the predicted survival curves for the 3-year and 5-year patients in the training and validation sets in Fig. 6 are closer to the actual curves, and the curves fit better, indicating that the model is more accurate.

#### Validation of nomogram in patients with high-grade astrocytoma

**Figure 7:**
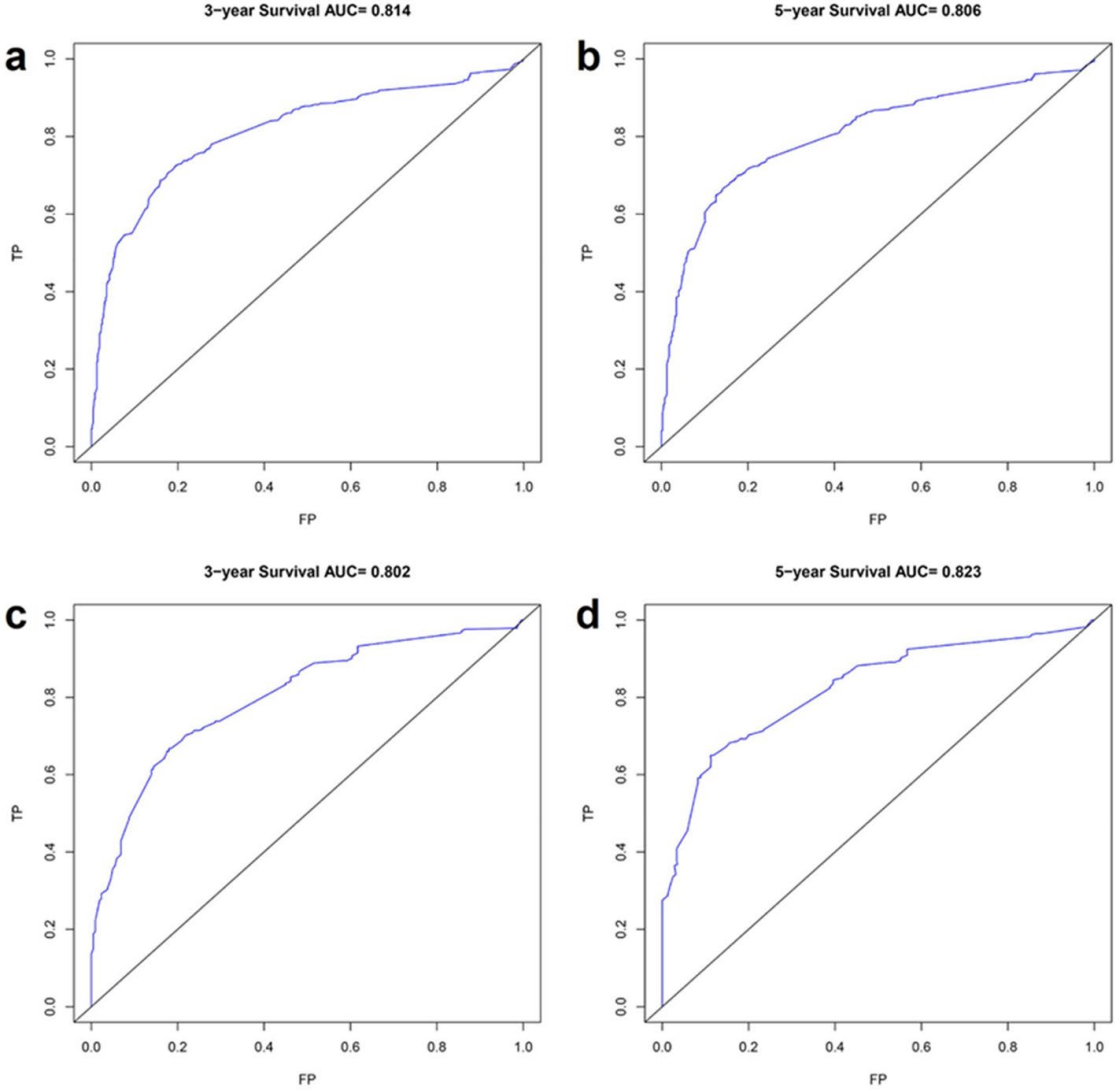
ROC curves of 3-year and 5-year survival prediction in patients with high-grade astrocytoma (**a** and **b** are the training set, **c** and **d** are the validation sets).

**Figure 8:**
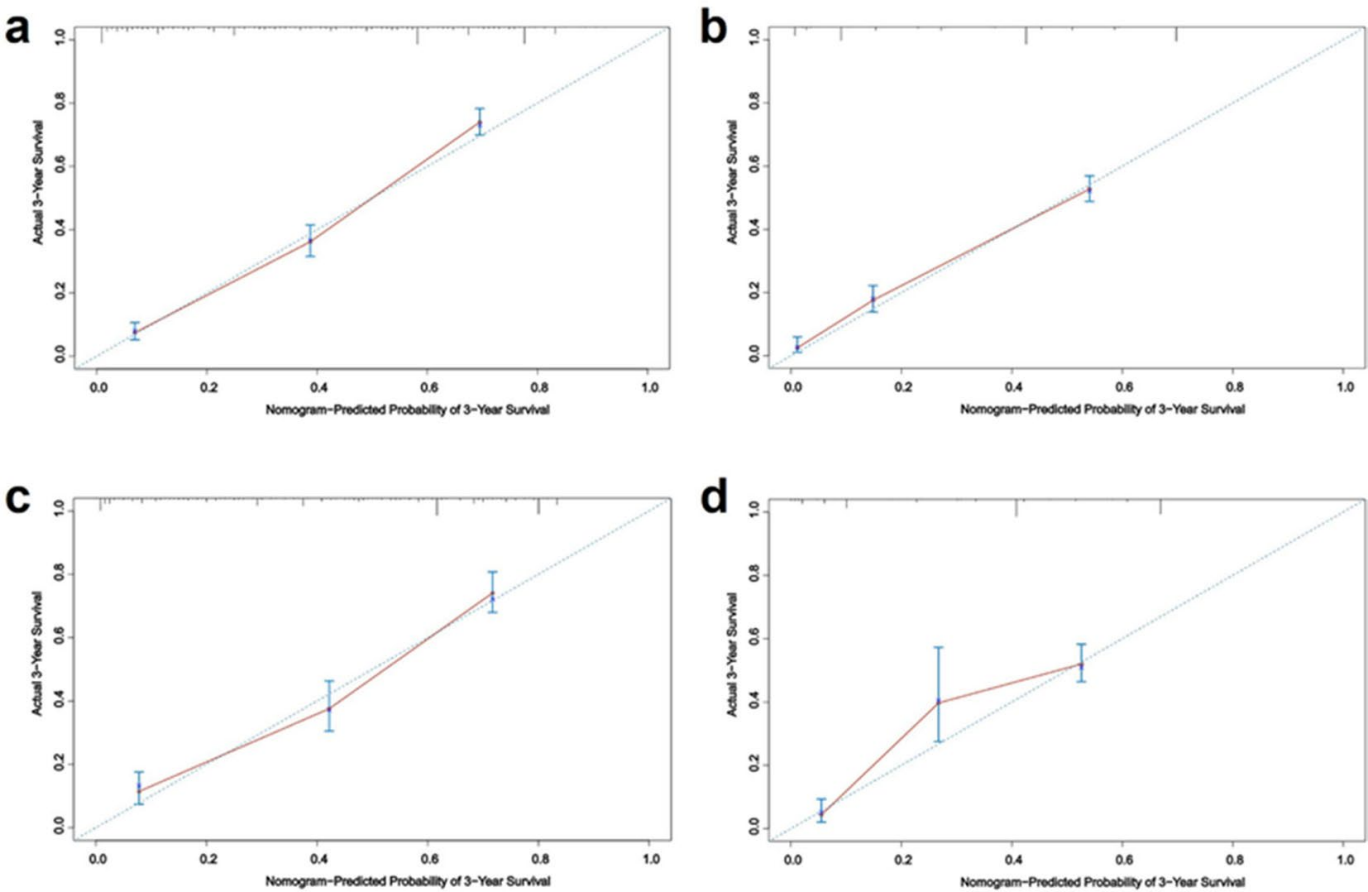
3-year and 5-year survival calibration curves for patients with high-grade astrocytoma (**a** and **b** are training sets, **c** and **d** are validation sets).

The AUC values of 3-year and 5-year survival rates of patients with high-grade astrocytoma training set were 0.814 and 0.806, respectively, and the AUC values of 3-year and 5-year survival rates of patients in the validation set were 0.802 and 0.823, respectively (Fig. 7). The C-index was 0.774 (95% CI 0.758, 0.790) for patients in the training set and 0.766 (95% CI 0.752, 0.780) for patients in the validation set. The 3-year and 5-year predicted survival curves of patients in the training and validation sets were in line with the true curves (Fig. 8).

## Discussion

Under the current trend of "digital medicine", it is important for both doctors and patients to use a combination of clinical diagnosis and intelligent means to determine the patient's condition and prognosis related risk factors. On the one hand, it can assist doctors to understand the patient's condition in time for more correct treatment; on the other hand, it is conducive to patients having a clearer understanding of their own conditions, which can greatly promote communication between doctors and patients. At the same time, in recent years, more and more scholars have conducted tumor research by mining SEER database, thus generating a variety of tumor prediction models, which may become a new direction for tumor research in the future^14^. Patients with astrocytoma of the brain diagnosed from 2004 to 2015 in the SEER database were included in this study, and a total of 2214 patients were screened according to the inclusion and exclusion criteria. Patients were randomly divided into training set and validation set according to different levels in a ratio of 7:3. Results from a univariate Kaplan–Meier survival curve analysis showed that the factors we included had an impact on patient survival, regardless of whether the tumor was low-grade or high-grade brain astrocytoma, with the exception of age and gender. The results of univariate and multifactor cox regression analysis of the training set data for patients of both grades showed that no radiotherapy and chemotherapy were protective factors for patients with low-grade brain astrocytoma with an OR less than 1, whereas the opposite was true for high-grade. It could indicate that patients with certain tumors of low grade would have longer survival without radiotherapy treatment, while patients with high-grade astrocytoma would need radiotherapy to survive longer. This result is clinically consistent and has some clinical significance. Meanwhile, the COX regression results affecting patient survival were consistent with the K-M curve, indicating the accuracy of the results.

Age has been found to be an important factor affecting the survival of patients in both low-grade and high-grade brain astrocytoma, and this result is more consistent with the findings of other scholars. Previous studies have also found a strong relationship between brain tumors and age^8^, older age predicts higher risk of disease^15,16^. However, some scholars studying advanced age and brain tumors have also found that elderly people may have slower tumor progression^17^, and low-grade and high-grade brain tumor log-rank test results and the Cox regression results indicated that older patients are more likely to have lower survival rates. In conclusion, age is an extremely important factor in the prognosis of patients with brain tumors and deserves further study. The gender distribution in this study was relatively balanced. In terms of racial distribution, Whites were overwhelmingly represented. In this study, the K-M curve and Cox regression results showed that the differences between sex and race were not statistically significant (P > 0.5). Studies have found that the incidence and mortality of brain tumors in both men and women have decreased year by year in recent years, but no significant differences have been found between sexes and races^18^.

The primary site of the patient's brain astrocytoma is also an important factor affecting survival. By comparing the K-M survival curves of low-grade brain astrocytoma with those of high-grade brain astrocytoma, we can find that the survival rate of patients with low-grade brain astrocytoma is significantly higher than that of high-grade brain astrocytoma, and this result is consistent with clinical reality. The data of this study has been analyzed to find that most of the tumors are concentrated in the cerebrum, and experts who have studied children's brain tumors have found that children's brain tumors, especially astrocytoma, are more common in the cerebellum^19^, which may be related to the wider distribution of age contained in the data of this study. Therefore, the age of the patient can affect the distribution of astrocytoma in the brain. We found that the survival rate of patients with pilocytic astrocytoma, a slow-growing benign tumor that generally does not require radiotherapy, is the highest among both low-grade and high-grade brain astrocytoma by K–M survival curves of brain tumor histology type. The results of cox regression showed that diffuse astrocytoma was a major risk factor for patient survival and astrocytoma has a poor prognosis^9,20^. At the same time, in this study, we found that the survival rate of patients with high-grade brain astrocytoma with bilateral tumors was lower than that of patients with unilateral tumors by K–M survival curves, and a greater number of tumors, deeper extension, and sequence number were associated with poorer patient survival. But this study found that the smaller the tumor, the lower the survival rate of patients, studies on breast cancer^21^, adult glioma^16^ and peripheral schwannoma^22^ have found that larger tumors are related to poor prognosis, the clinical inconsistency may be due to the fact that the classification of astrocytic tumors in this study is not the latest classification standard, and there are no molecular typing-related classification standards in the 2004–2015 database.. Current treatment for high-grade brain tumors or malignant brain tumors^23^, surgery on patients, and simultaneous radiotherapy and chemotherapy can benefit the survival of patients^7,14^. The results of this study yielded an OR greater than 1 for both low-grade and high-grade tumors in patients without surgery relative to patients with surgery, indicating that surgery has a better prognosis for patients, and this result is consistent with the current conventional treatment of brain tumors in clinical practice. The present study also has some limitations, as the SEER database itself provides a limited amount of information, and the database does not provide any information on genes, so we could not study the prognostic factors of brain tumors at the genetic level^19^. Second, with the development of gene sequencing, brain tumors have entered the era of molecular typing, the data extracted in this study before 2016, there was no molecular typing in the database, so molecular typing analysis could not be performed, and different histotypings would change the prognosis of patients, and it is worth further research in the future.

In conclusion, In this study, the risk factors for patients with low-grade and high-grade brain astrocytoma were screened by univariate Kaplan–Meier survival curves, respectively, while the risk factors affecting the prognosis of patients with brain astrocytoma in both grades were more completely included and the nomogram were successfully established, with high AUC and C-index in both tumor training and validation sets for both grades, and a good calibration curve fit, indicating that the nomogram has a strong predictive ability to predict the 3- year and 5-year survival rates of patients. However, since the data were obtained from the United States, more studies are needed to verify whether the results obtained from the application of this data can be applied to the Chinese population, and the results obtained from this study can provide some reference for clinicians.

## Data Availability

The data that support the findings of this study are available from SEER database but restrictions apply to the availability of these data, which were used under license for the current study (ID: 12533-Nov2021), and so are not publicly available. Data are however available from the authors upon reasonable request and with permission of SEER database.
